# The sensitivity and specificity of the neurological examination in polyneuropathy patients with clinical and electrophysiological correlations

**DOI:** 10.1371/journal.pone.0171597

**Published:** 2017-03-01

**Authors:** Alon Abraham, Majed Alabdali, Abdulla Alsulaiman, Hana Albulaihe, Ari Breiner, Hans D. Katzberg, Danah Aljaafari, Leif E. Lovblom, Vera Bril

**Affiliations:** 1 Ellen and Martin Prosserman Centre for Neuromuscular Diseases, Division of Neurology, Department of Medicine, University Health Network, University of Toronto, Toronto, Canada; 2 Department of Neurology, King Fahad Hospital of the University, University of Dammam, Dammam, Saudi Arabia; 3 Department of Neurology, King Khalid University Hospital, King Saud University, Riyadh, Saudi Arabia; 4 Division of Endocrinology and Metabolism, Department of Medicine, Mount Sinai, Hospital and Lunenfeld Tanenbaum Research Institute, University of Toronto, Toronto, Canada; Hirosaki Daigaku, JAPAN

## Abstract

**Introduction:**

Polyneuropathy is one of the most prevalent neurologic disorders. Although several studies explored the role of the neurological examination in polyneuropathy, they were mostly restricted to specific subgroups of patients and have not correlated examination findings with symptoms and electrophysiological results.

**Objectives:**

To explore the sensitivity and specificity of different neurological examination components in patients with diverse etiologies for polyneuropathy, find the most sensitive combination of examination components for polyneuropathy detection, and correlate examination findings with symptoms and electrophysiological results.

**Methods:**

Patients with polyneuropathy attending the neuromuscular clinic from 01/2013 to 09/2015 were evaluated. Inclusion criteria included symptomatic polyneuropathy, which was confirmed by electrophysiological studies. 47 subjects with no symptoms or electrophysiological findings suggestive for polyneuropathy, served as controls.

**Results:**

The total cohort included 312 polyneuropathy patients, with a mean age of 60±14 years. Abnormal examination was found in 95%, most commonly sensory findings (86%). The most common abnormal examination components were impaired ankle reflexes (74%), vibration (73%), and pinprick (72%) sensation. Combining ankle reflex examination with vibration or pinprick perception had the highest sensitivity, of 88%. The specificities of individual examination component were generally high, excluding ankle reflexes (62%), and vibration perception (77%). Abnormal examination findings were correlated with symptomatic weakness and worse electrophysiological parameters.

**Conclusion:**

The neurological examination is a valid, sensitive and specific tool for diagnosing polyneuropathy, and findings correlate with polyneuropathy severity. Ankle reflex examination combined with either vibration or pinprick sensory testing is the most sensitive combination for diagnosing polyneuropathy, and should be considered minimal essential components of the physical examination in patients with suspected polyneuropathy.

## Introduction

Despite technological advances, the neurologic evaluation remains first and foremost a bedside exercise[1]. The clinical history and the neurologic examination play an important role in the diagnosis of various nervous system diseases. The crystallization of an accurate clinical picture regarding localization, is unique for the field of neurology, and does not occur to the same degree in any other branch of medicine[1].

Polyneuropathy is one of the most prevalent neurologic disorders, with an overall prevalence of 2.4%, increasing to 8% in people older than 55 years[2]. Polyneuropathy most commonly presents with sensory symptoms, occasionally accompanied by weakness, typically in a distal symmetric distribution. However, symptoms alone have a relatively poor diagnostic accuracy in predicting the presence of polyneuropathy, and the most accurate diagnosis is made by a combination of neuropathic symptoms and signs, and electrophysiological findings[3]. Nonetheless, patients with polyneuropathy restricted to small nerve fibers, have minimal findings on the neurological examination, such as reduced pinprick or temperature sensation, and normal nerve conduction studies, making the diagnosis of small fiber neuropathy challenging[4].

The neurological examination is relatively inexpensive, but nonetheless may reveal more than the most expensive laboratory tests and imaging studies. However, the examination is more art than science, as limited evidence supports its value, and therefore additional research has been recommended[5]. Previous studies have shown excessive variability and over-diagnoses of signs,[6], which improves by using unequivocally abnormal signs and symptoms, and taking age, sex, and physical variables into account.[7] Although several studies have explored the role of the neurological examination in polyneuropathy, they were mostly restricted to specific subgroups of patients, most frequently those with diabetic polyneuropathy, and have not explored the correlation between examination findings and symptoms and electrophysiological results[3].

The purpose of this study was to explore the sensitivity and specificity of different neurological examination components in patients with diverse etiologies for polyneuropathy, and to find the most sensitive combination of examination components which could be used to screen for polyneuropathy. In addition, we aimed to correlate examination findings with symptoms and electrophysiological results, in order to determine whether neurologic examination findings also correlate with polyneuropathy severity.

## Materials and methods

In this study, we extracted the demographic data, clinical history, and neurological and electrophysiological findings of 312 patients diagnosed with polyneuropathy. All patients attended the Prosserman Family Neuromuscular clinic, Toronto General Hospital, University Health Network from January 2013 to September 2015. In addition, 47 control subjects, attending the clinic from February 2015 to August 2016, were examined. The Research Ethics Board of the University Health Network approved the current study protocol and waived informed consent for polyneuropathy patients, which were studied retrospectively. All control subjects were recruited prospectively as part of another study evaluating patients with 50 or more years of type I diabetes duration, for future biomarker studies, and provided informed consent.

Inclusion criteria for patients with polyneuropathy included a diagnosis of polyneuropathy in symptomatic patients who had confirmation by electrophysiological findings (reference standard). Patients with mononeuropathies or pure small fiber neuropathy were excluded from this study. The reason for exclusion of patients with small fiber neuropathy was the relatively nonspecific presentation, combined with minor or no neurological findings and normal nerve conduction studies, making a definitive diagnosis challenging[4]. Although small fiber neuropathy can be confirmed by skin punch biopsy[8], or other specialized tests for small nerve fiber function[9], those were not assessed in this study. In addition, the minimal neurological examination findings and the normal nerve conduction studies do not allow correlation of examination findings with symptoms and nerve conduction studies, so that this patient subgroup is not relevant for the current study objectives.

Control subjects were age- and gender- matched with type 1 diabetes patients from a different study. Exclusion criteria included the presence of diabetes, or symptoms or electrophysiological evidence suggestive for polyneuropathy. Specifically, subjects with a clinical history of sensory symptoms, ataxia, or weakness were excluded. In addition, subjects with a sural sensory nerve action potential (SNAP) amplitude <6 μV, compound muscle action potential (CMAP) amplitude of the peroneal nerve <2 mV, or motor or sensory nerve conduction velocities <40 m/s, were excluded[10]. As the clinical evaluation of control subjects was performed using the Toronto Clinical Neuropathy Scale (TCNS)[11], the neurological examination was limited to lower limb reflexes, and detailed sensory examination of the lower limbs.

All patients underwent detailed comprehensive neurological examination by neuromuscular experts or fellows, and nerve conduction studies. Neurological examination findings included proximal or distal muscle weakness, biceps, triceps, brachioradialis, knee and ankle reflexes, sensation for vibration, pinprick, temperature, light touch and proprioception, gait and tandem gait. For the purposes of this study, the presence of any degree of impairment on examination was considered as abnormal. Specifically, abnormal examination findings were defined as any degree of weakness, decreased or absent reflexes, decreased or absent sensation for each sensory modality, and any gait abnormality. Nerve conduction study findings for the sural and peroneal nerves were recorded. Nerve conduction studies were performed using the Sierra Wave instrument (Cadwell Laboratories Inc., Kennewick, WA, USA), using surface stimulating and recording techniques according to the standards of the Canadian Society of Clinical Neurophysiology and the American Association of Neuromuscular and Electrodiagnostic Medicine.[12,13]. Peroneal and sural CMAP and SNAP amplitudes were measured from baseline to positive peak. Limb temperature was measured prior to nerve conduction studies, and if required, warming was performed to ensure a surface temperature of >32°C in the hands and >31°C in the feet. All patients had at least one electrophysiological abnormality of either the sural or peroneal nerves, which are considered to be the most sensitive for detecting a distal symmetric polyneuropathy[3]. An abnormal test was defined by: reduced sural SNAP amplitude, reduced CMAP amplitude in the extensor digitorum brevis muscle after peroneal nerve stimulation, increased distal sensory or motor latency latencies, or reduced motor or sensory nerve conduction velocities. Demographic, clinical and electrophysiological findings were compared between patients with common etiologies for polyneuropathy, and between patients with polyneuropathy and control subjects. The sensitivity and specificity of various individual and combined components of the neurological examination were calculated. In addition, the sural SNAP and peroneal CMAP amplitudes were compared between patients with normal and abnormal neurological examination components (e.g. impaired vibration perception or ankle reflexes or distal weakness), and between patients with a different number (range 0–4) of abnormal examination components, out of vibration perception, distal strength, ankle reflexes and gait.

### Statistical analysis

Clinical and electrophysiological characteristics were expressed as means ± standard deviations (SD) for continuous data or as frequency and percent for ordinal data. Comparisons between patients with polyneuropathy and controls, and between symptoms and electrophysiological findings in patients with different numbers of abnormal examination findings were made using the χ2 test and analysis of variance (ANOVA). Comparisons between nerve action potential amplitudes was performed using the t-test. Point biserial correlation coefficients were calculated for nerve action potential amplitudes and examination findings. Significance was set at α-level of 0.05.

## Results

The total cohort included 312 patients diagnosed with polyneuropathy (S1 Dataset). The most common aetiologies included diabetes mellitus (33%), idiopathic (19%), prediabetes (14%), and chronic inflammatory demyelinating polyneuropathy (CIDP) (13%), followed by various other aetiologies: vitamin deficiency, genetic, toxic, and inflammatory (Table 1).

**Table 1:** Distribution of common aetiologies in 312 patients with polyneuropathy.

Aetiology	n (%)
Diabetes	103 (33)
Idiopathic	58 (19)
Prediabetes	43 (14)
CIDP	41 (13)
B12 deficiency	17 (8)
CMT	23 (7)
Chemotherapy	14 (4)
Alcohol	12 (4)
Anti MAG	9 (3)
Renal	7 (2)
HNPP	6 (2)
GBS	5 (2)
MADSAM	5 (2)
Vasculitis	5 (2)

CIDP—Chronic inflammatory demyelinating polyneuropathy; CMT—Charcot-Marie-Tooth; MAG—Myelin associated glycoprotein; HNPP—Hereditary neuropathy with liability to pressure palsies; GBS—Guillain-Barre Syndrome; MADSAM—Multifocal acquired demyelinating sensory and motor neuropathy

The mean age of all patients was 60±14 years, comprising 31% females. Commonly reported comorbidities and symptoms are shown in Table 2.

**Table 2:** Demographics, comorbidities and neurological symptoms of 312 patients with polyneuropathy.

	Total Cohort (n = 312)
Age (years)	60±14
Female, n (%)	96 (31%)
Diabetes, n (%)	93 (30%)
Pre-diabetes, n (%)	6 (3%)
Hypertension, n (%)	135 (44%)
Thyroid Disease, n (%)	29 (9%)
Smoking, n (%)	53 (17%)
Alcohol, n (%)	34 (11%)
**Symptoms**	
Sensory, n (%)	263 (88%)
Proximal, n (%)	27 (9%)
Distal, n (%)	258 (86%)
**Pain**, n (%)	138 (49%)
**Weakness**	
Proximal, n (%)	60 (22%)
Distal, n (%)	105 (38%)

The most common symptoms included distal sensory symptoms (86%), followed by neuropathic pain (49%) and distal muscle weakness (38%). However, the most common symptoms in patients without sensory manifestations (14%), were muscle weakness (69%), followed by pain (35%), and muscle cramps and gait instability (11%) (data not shown).

The neurological examination showed at least one abnormal finding in 95% of patients. The most common abnormality was impaired ankle reflexes (74%), followed by impaired vibration perception (73%) and pinprick sensation (72%). The least sensitive components included proximal strength (18%), followed by proprioception (36%) and gait abnormality (43%). Impaired vibration perception was most common in DSP and CIDP (81% and 88% respectively), and least common in CMT (70%). Impaired ankle reflexes were most common in CMT (96%), and the least common for idiopathic polyneuropathy (55%). Distal weakness was the most common in CIDP (76%) and CMT (74%), and the least common for idiopathic polyneuropathy (27%). Proximal weakness was most common in CIDP (41%) (Table 3).

**Table 3:** Abnormal neurological examination findings in patients with common types of polyneuropathy.

	Total Cohort (n = 312)	DSP (n = 103)	Idiopathic (n = 58)	Pre-DM (n = 43)	CIDP (n = 41)	CMT (n = 23)
**Weakness**						
Proximal	18%	21%	7%	5%	41%	4%
Distal	44%	38%	27%	41%	76%	74%
**Impaired reflexes**						
Upper limbs	44%	57%	23%	43%	68%	70%
Knee	52%	61%	34%	54%	68%	65%
Ankle	74%	86%	55%	72%	85%	96%
**Sensory deficits**						
Vibration	73%	81%	74%	60%	88%	70%
Pinprick	72%	81%	71%	71%	88%	59%
Temperature	60%	79%	57%	47%	71%	47%
Light touch	45%	68%	36%	20%	53%	30%
Proprioception	36%	39%	30%	33%	48%	48%
**Any abnormality**	95%	98%	88%	98%	100%	100%

Various combinations of any 2 components of the neurological examination, with at least one abnormality, showed the highest sensitivity at 88% for combining ankle reflexes with vibration or pinprick perception, followed by the combination of vibration perception with distal strength testing of 83% (Table 4).

**Table 4:** Sensitivities for different combinations of abnormal neurological examination findings with at least one abnormal component, in 312 patients.

	Vibration	Pinprick	Temperature	Light touch	Proprioception	Ankle reflexes
Pinprick	83%					
Temperature	80%	76%				
Light touch	78%	75%	64%			
Proprioception	75%	74%	64%	56%		
Ankle Reflexes	88%	88%	84%	82%	78%	
Weakness *	83%	82%	75%	68%	59%	79%

* Distal weakness

Patients with polyneuropathy and control subjects had similar ages (63±14 vs. 60±7 years respectively; p = 0.11). However, the gender distribution differed (31% vs. 53% females; p<0.01) (Data not shown). The most specific examination component for polyneuropathy was found to be proprioception perception (98%), followed by light touch sensation and knee reflexes (96%). The least specific component was found to be ankle reflexes (62%), followed by vibration perception (77%) (Table 5). Specificity determination was limited to lower limb reflexes, and detailed sensory examination of the lower limbs, as other neurological examination components were not studied in control subjects.

**Table 5:** Comparison of abnormal neurological examination findings between patients and controls.

	Total Cohort (n = 312)	Controls (n = 47)	Specificity	PPV	NPV
**Impaired reflexes**					
Knee	52%	4%	96%	99%	24%
Ankle	74%	38%	62%	92%	27%
**Sensory deficits**					
Vibration	73%	23%	77%	95%	31%
Pinprick	72%	9%	91%	98%	34%
Temperature	60%	11%	89%	97%	27%
Light touch	45%	4%	96%	98%	23%
Proprioception	36%	2%	98%	99%	20%

PPV–Positive Predictive Value; NPV–Negative Predictive Value. p<0.0001 for each comparison between groups.

Abnormal electrophysiological testing was present in all patients, with the most common finding being reduced sural SNAP amplitude in 91% of the total cohort. Mean sural SNAP or peroneal CMAP amplitudes showed weak correlations with most examination components, and were lower in patients with abnormal neurological examination findings, for each examination component, excluding proximal weakness (Table 6).

**Table 6:** Comparison of sural SNAP and peroneal CMAP potential amplitudes between normal and abnormal exam components, and correlations between amplitudes and various exam components.

	Sural SNAP Amplitudes (μV)				Peroneal CMAP Amplitudes (mV)			
	r	N	Ab	p	r	N	Ab	p
**Weakness**								
Proximal	0.08	2.6 ± 3.4	3.4 ± 6.1	0.15	0.08	1.9 ± 1.9	1.5 ± 1.6	0.15
Distal	0.06	2.9 ± 3.7	2.5 ± 4.5	0.34	0.36	2.4 ± 1.9	1 ± 1.6	**<0.01**
**Impaired reflexes**								
Upper limbs	0.09	3 ± 4.3	2.3 ± 3.6	0.13	0.19	2.1 ± 1.8	1.4 ± 1.8	**<0.01**
Knee	0.13	3.3 ± 4.5	2.2 ± 3.5	**<0.05**	0.26	2.3 ± 1.9	1.3 ± 1.7	**<0.01**
Ankle	0.21	4.2 ± 5.4	2.2 ± 3.3	**<0.01**	0.37	3 ± 1.9	1.4 ± 1.7	**<0.01**
**Sensory deficits**								
Vibration	0.29	4.7 ± 6.1	2 ± 2.6	**<0.01**	0.25	2.6 ± 2	1.5 ± 1.7	**<0.01**
Pinprick	0.22	4.2 ± 6.1	2.2 ± 2.7	**<0.01**	0.17	2.3 ± 2.1	1.6 ± 1.7	**<0.01**
Temperature	0.25	3.9 ± 5.4	1.9 ± 2.4	**<0.01**	0.19	2.2 ± 2	1.5 ± 1.7	**<0.01**
Light touch	0.18	3.3 ± 4.9	1.9 ± 2.4	**<0.01**	0.08	1.9 ± 1.8	1.6 ± 1.8	0.19
Proprioception	0.27	3.6 ± 4.6	1.3 ± 2.2	**<0.01**	0.23	2.1 ± 1.9	1.3 ± 1.7	**<0.01**
Gait	0.21	3.5 ± 4.7	1.8 ± 2.9	**<0.01**	0.29	2.3 ± 1.9	1.2 ± 1.7	**<0.01**
Tandem	0.21	3.8 ± 4.4	2 ± 3.7	**<0.01**	0.34	2.5 ± 1.9	1.3 ± 1.6	**<0.01**

Data presented as mean ± Standard deviation. Statistically significant p values (<0.05) are bolded. N–Normal; Ab–Abnormal

Patients with a higher number of abnormal neurological examination components (range 0–4), including vibration perception, distal strength, and gait, had a higher frequency of symptomatic weakness, lower sural SNAP and peroneal CMAP amplitudes, and lower peroneal motor nerve conduction velocities (table 7).

**Table 7:** Comparison of symptoms and nerve conduction study results, between patients with various number of abnormal exam components, including vibration perception, distal strength, ankle reflex and gait.

Abnormal Exam components	0 (n = 21)	1 (n = 57)	2 (n = 79)	3 (n = 65)	4 (n = 65)	P value
**Symptoms**						
Sensory (%)	80	88	90	89	78	0.80
Weakness (%)	20	24	26	63	84	**<0.0001**
Pain (%)	35	49	48	57	53	0.22
**Nerve conduction studies**						
Sural						
Amplitude (mV)	5.4 ± 5.3	4.1 ± 6.3	2.8 ± 3	2.3 ± 2.9	1.3 ± 2.7	**<0.0001**
CV (m/s)	41 ± 5	42 ± 5	41 ± 4	40 ± 5	40 ± 6	0.14
Peroneal						
Amplitude (mV)	4 ± 1.6	2.6 ± 1.8	2 ± 1.6	1.3 ± 1.8	0.8 ± 1.3	**<0.0001**
CV (m/s)	41 ± 4	39 ± 7	37 ± 8	36 ± 7	30 ± 10	**<0.0001**

Statistically significant p values (<0.05) are bolded.

CV–Conduction velocity

## Discussion

The neurological examination is a fundamental part of the evaluation of patients with central and peripheral nervous system disorders, that along with history, and other additional tests, such as laboratory, electrophysiological, and radiological tests, allow formulation of a diagnosis. Our study demonstrates the high sensitivity and specificity of the neurological examination in polyneuropathy detection, and the validity of the examination is reinforced by the relationship with clinical symptoms and nerve conduction study findings. In addition to the diagnostic role of the neurological examination, the burden of abnormal findings correlates with polyneuropathy severity, as reflected by the higher frequency of symptomatic weakness and worse electrophysiological parameters.

The most common abnormal components of the neurological examination in patients with polyneuropathy from diverse aetiologies, included decreased or absent ankle reflexes (74%), impaired vibration perception (73%) and reduced pinprick sensation (72%). In contrast, strength and gait examination were the least sensitive (44% and 43% respectively). These findings are not surprising considering the fact that most forms of peripheral neuropathy are clinically sensory predominant[14], as was also shown in our study, with 86% of patients having distal sensory symptoms, compared to 38% of patients having distal symptomatic weakness (Table 2). As most peripheral neuropathies are length dependent, distal muscle weakness may be masked by unaffected proximal muscles performing synergistic functions, in contrast to the lack of a similar backup system for sensory activity[14].

Combining ankle reflex examination with vibration or pinprick sensory examination, had the highest sensitivity for polyneuropathy detection, at 88% (Table 4), and therefore should be an integral part of the physical examination in cases of suspected polyneuropathy in any clinic. The high sensitivity of combined ankle reflex and vibration perception examination might be predicted as both are large fiber functions, and the reference standard of nerve conduction studies is a large fiber measure. A similar sensitivity of the combined ankle reflex and pinprick sensory examination is more surprising, and might be explained by the the fact that these examination components appear to be the most sensitive markers of large and small fiber functions respectively, and that most neuropathies are mixed in type[15]. Nonetheless, it should be noted that ankle reflex and vibration perception examinations were also found to be the least specific (Table 5), most likely due to changes related to aging, underscoring the importance of considering age when performing the neurological examination, and integrating the full clinical picture. In patients with diabetic polyneuropathy, the most common type of polyneuropathy in our cohort, as well as worldwide[16–18], the sensitivity of most examination components was even higher than in to the total cohort, possibly due to a more severe neuropathy associated with more frequent neurological examination abnormalities. Similarly, patients with CIDP in this cohort had sensory impairments with equal frequency to those with diabetic polyneuropathy, and in additional, demonstrated the highest frequency of proximal and distal muscle weakness, again confirming the higher sensitivity of the neurological examination for this treatable polyneuropathy. Interestingly, vibration perception previously described to correlate with treatment responsiveness in CIDP, demonstrated the importance of this sensory test in what is considered often to be primarily a motor polyneuropathy [19]. Patients with hereditary polyneuropathy were exceptional, being the only subgroup with motor predominant signs, while patients with idiopathic polyneuropathy had the least frequent neurological signs, in line with previous literature, perhaps due to relatively mild polyneuropathy (Table 3)[10]. These findings therefore suggest that the neurological examination can provide a clue on etiology in certain cases.

The sural SNAP and peroneal CMAP amplitudes reflect the number of sensory and motor nerve fibers in the lower limbs, and are considered to be the most sensitive and reliable for detecting a distal symmetric polyneuropathy[3]. Our study results demonstrate lower sural SNAP and peroneal CMAP amplitudes for the majority of examination components (Table 6), as well as worse electrophysiological parameters with an increasing number of abnormal clinical findings (Table 7). As expected, sensory deficits tended to correlate better with sural SNAP amplitudes, while weakness, reflexes, and gait, tended to correlate better with peroneal CMAP amplitudes. These results indicate that in addition to diagnostic utility, the neurological examination correlates with polyneuropathy severity, as measured by electrophysiological indices, which is an expected finding considering the progressive axonal loss as polyneuropathies progress. The lack of amplitude differences between patients with and without proximal weakness, is most likely explained by the fact that routine nerve conduction studies most commonly evaluate distal nerve segments (e.g. sural and peroneal). Nonetheless, the weak correlation between the examination components and the electrophysiological findings, suggests a lack of significant overlap, and a unique contribution by each assessment technique. The cumulative number of abnormal neurological examination findings correlated with the frequency of symptomatic limb muscle weakness, but not with sensory symptoms or pain. This finding may be explained on the basis that sensory examination forms a smaller part of the overall neurological examination, than motor assessment, and thus the relationship with motor deficits is more apparent. Furthermore, the absence of a correlation between pain and polyneuropathy severity has been described previously [20].

Our study has a few limitations. In this retrospective study, examiners were not blinded to the patients’ clinical history, reason for referral and suspected diagnosis. Therefore, overestimating neurological signs, which has been described previously[6], cannot be excluded. In addition, we studied routine clinical examination findings, which are not restricted to unequivocally abnormal signs, and we have not taken into account age, sex, and physical variables, which have shown to improve physician proficiency compared with simple elicitation of signs and symptoms[7]. As the study cohort included patients from a tertiary center, a referral bias may exist, leading to misrepresentation of different types of peripheral neuropathies. Nonetheless, DSP and idiopathic neuropathy were the most common etiologies, in above 50% of patients, similar to a report from a setting of community neurologists[21]. In addition, this study included patients with symptomatic and electrophysiologically confirmed polyneuropathy, excluding those with pure small fiber neuropathy. Thus, we cannot draw conclusions for this specific type of polyneuropathy, in which typically there are minimal findings on the neurological examination. In addition, in order to optimize the accuracy of polyneuropathy diagnosis for the purpose of this study, we chose to include patients with polyneuropathy confirmed by at least one abnormal parameter on electrophysiological testing and did not use the more rigorous case definition for distal symmetric polyneuropathy, as suggested by England at el.[3]. Finally, although specificity was addressed using controls with no symptoms or electrophysiological evidence for polyneuropathy, we did not explored specificity using patients with various other neuromuscular disorders, in order to assess specificity more broadly. In addition, specificity determination was limited to lower limb tests, and did not refer to other components of the neurological examination, and reproducibility was not assessed[22].

## Supporting information

S1 Dataset (XLSX)
